# Functional Genomics Via Metabolic Footprinting: Monitoring Metabolite Secretion by *Escherichia Coli* Tryptophan Metabolism Mutants Using FT–IR and Direct Injection Electrospray Mass Spectrometry

**DOI:** 10.1002/cfg.302

**Published:** 2003-07

**Authors:** Naheed N. Kaderbhai, David I. Broadhurst, David I. Ellis, Royston Goodacre, Douglas B. Kell

**Affiliations:** 1 Institute of Biological Sciences University of Wales Aberystwyth Wales Ceredigion SY23 3DD UK; 2 Department of Chemistry UMIST Faraday Building Sackville Street Manchester M60 1QD UK

## Abstract

We sought to test the hypothesis that mutant bacterial strains could be discriminated from each other on the basis of the metabolites they secrete into the medium (their ‘metabolic footprint’), using two methods of ‘global’ metabolite analysis (FT–IR and direct injection electrospray mass spectrometry). The biological system used was based on a published study of *Escherichia coli* tryptophan mutants that had been analysed and discriminated by Yanofsky and colleagues using transcriptome analysis. Wild-type strains supplemented with tryptophan or analogues could be discriminated from controls using FT–IR of 24 h broths, as could each of the mutant strains in both minimal and supplemented media. Direct injection electrospray mass spectrometry with unit mass resolution could also be used to discriminate the strains from each other, and had the advantage that the discrimination required the use of just two or three masses in each case. These were determined via a genetic algorithm. Both methods are rapid, reagentless, reproducible and cheap, and might beneficially be extended to the analysis of gene knockout libraries.


**Comparative and Functional Genomics**
*Comp Funct Genom* 2003; **4**: 376–391.
Published online 18 July 2003 in Wiley InterScience (www.interscience.wiley.com). **DOI:** 10.1002/cfg.302

**Primary Research Paper**

### Functional genomics via metabolic footprinting: monitoring metabolite secretion by *Escherichia coli* tryptophan metabolism mutants using FT–IR and direct injection electrospray mass spectrometry

Naheed N. Kaderbhai, David I. Broadhurst, David I. Ellis, Royston Goodacre# and Douglas B. Kell*,#
*Institute of Biological Sciences, University of Wales, Aberystwyth, Ceredigion SY23 3DD, Wales, UK*

**Correspondence to:
Douglas B. Kell, Department of Chemistry, UMIST, Faraday Building, Sackville Street, Manchester M60 1QD, UK.
E-mail: dbk@umist.ac.uk*

*#Present address: Department of Chemistry, UMIST, Faraday Building, Sackville Street, Manchester M60 1QD, UK.*

Received: 15 January 2003
Revised: 23 April 2003
Accepted: 22 May 2003

### Abstract

**We sought to test the hypothesis that mutant bacterial strains could be discriminated from each other on the basis of the metabolites they secrete into the medium (their 'metabolic footprint'), using two methods of 'global' metabolite analysis (FT–IR and direct injection electrospray mass spectrometry). The biological system used was based on a published study of *Escherichia coli* tryptophan mutants that had been analysed and discriminated by Yanofsky and colleagues using transcriptome analysis. Wild-type strains supplemented with tryptophan or analogues could be discriminated from controls using FT–IR of 24 h broths, as could each of the mutant strains in both minimal and supplemented media. Direct injection electrospray mass spectrometry with unit mass resolution could also be used to discriminate the strains from each other, and had the advantage that the discrimination required the use of just two or three masses in each case. These were determined via a genetic algorithm. Both methods are rapid, reagentless, reproducible and cheap, and might beneficially be extended to the analysis of gene knockout libraries. Copyright © 2003 John Wiley & Sons, Ltd.**

**Keywords:** **metabolome; metabolomics; metabolic footprinting; mass spectrometry; FTIR spectroscopy; *Escherichia coli*; tryptophan; genetic algorithm**

### Introduction

The systematic and complete genome sequencing of many organisms, including *Escherichia coli* (Blattner *et al*., 1997, 1998; Liang *et al*., 2002; Riley and Serres, 2000), brings the need to establish the cellular functions of all the genes, including the many novel genes, thereby uncovered (Brent, 1999, 2000; Clare and King, 2002; Hieter and Boguski, 1997; Kell and King, 2000; King *et al*., 2000; Oliver, 1996; Skolnick *et al*., 2000). Typical strategies include expression profiling at the level of the transcriptome (Burge, 2001; Devaux *et al*., 2001; Jurgen *et al*., 2001; Oshima *et al*., 2002; Tjaden *et al*., 2002) and the proteome (Akashi and Gojobori, 2002; Auer *et al*., 1998; Champion *et al*., 2001; Choe *et al*., 1998; Dunn, 1998; Futcher *et al*., 1997, 1999; Han *et al*., 2001; Joubert-Caron and Caron, 1999; Jurgen *et al*., 2001; Kabir and Shimizu, 2001; Loo *et al*., 2001; Thomas, 1999) and, given the importance of metabolism to cellular physiology (Cornish-Bowden and Cardenas, 2000; Michal, 1999), at the level of metabolism. With the emergence of many useful genomics-derived databases of metabolism (e.g. Goryanin *et al*., 1999; Kanehisa and Goto, 2000; Karp *et al*., 1996, 2002a, 2000b; Mendes, 2002; Ouzounis and Karp, 2000; Schilling and Palsson, 2000; Tomita *et al*., 1999; Wixon and Kell, 2000), experimental

Copyright © 2003 John Wiley & Sons, Ltd.

analysis of the metabolome (Kell and Mendes, 2000; Oliver, 1996, 1997; Oliver *et al.*, 1998; Raamsdonk *et al.*, 2001; ter Kuile and Westerhoff, 2001), the complement of low molecular weight metabolites in a cell (Covert *et al.*, 2001; Fiehn, 2001, 2002; Fiehn *et al.*, 2000, 2001; Hall *et al.*, 2002; Kose *et al.*, 2001; Roessner *et al.*, 2001; Taylor *et al.*, 2002) is now being seen as another essential step for improving our understanding of the living cell. In order to study these metabolites a number of factors have to be considered, as the changes that take place will be multiple, will affect molecules with very different chemistries, and there will be both intracellular and extracellular changes (e.g. Kramer, 1994). Their quantification, detection and identification using new technologies and methodologies provide both hurdles and opportunities.

Nuclear magnetic resonance (NMR) spectroscopy has been used for determination of *in vivo* metabolite levels in intact cells and cell extracts (Nicholson *et al.*, 1999; Raamsdonk *et al.*, 2001; Warne *et al.*, 2000) but the low sensitivity of the method in most laboratories restricts its use (Hartbrich *et al.*, 1996). More recently, a novel NMR spectroscopic approach to the direct biochemical characterization of bacterial culture broths was presented (Abel *et al.*, 1999). Low molecular weight organic components of broth supernatants from cultures of *Streptomyces citricolor* were analysed using one- (1D) and two-dimensional (2D) $^1$H-NMR spectroscopic methods; it was possible to identify and monitor simultaneously a range of media substrates and excreted metabolites which included 2-phenylethylamine, trehalose, succinate, acetate, uridine and aristeromycin. Signals were extensively overlapped in the $^1$H-NMR spectra of the whole broth mixtures, so directly coupled HPLC–NMR spectroscopy was also applied to the analysis of broth supernatants to aid spectral assignments. Multiple bond correlation for structural elucidation and peak assignments of individual components was also conducted using 2D NMR methods based on $^1$H–$^1$H and $^1$H–$^{13}$C correlations. This work showed that high-resolution NMR spectroscopic methods could provide a rapid and efficient means of investigating microbial metabolism directly without invasive or destructive sample pretreatment. van Eijk developed a high-throughput screening method using LC–MS (Caceres *et al.*, 2000; van Eijk *et al.*, 1999) to study the amino acids, in which the simultaneous application (and thus measurement) of multiple amino acid tracers was used coupled to liquid chromatography and mass spectrometry, resulting in the measurement of both the concentration and isotope enrichment of *O*-phthaldialdehyde (OPA)-derivatized plasma amino acids in one run. Considering the easier and cheaper derivatization procedure and instrumentation, the simultaneous collection of isotopomeric distribution spectra (enabling the application of multiple labelled components) and concentration data, the method presents an attractive alternative to traditional GC–MS applications for amino acids. Therefore, combining liquid chromatography with electrospray mass spectrometry (LC–ESI–MS) methods is attractive (Buchholz *et al.*, 2001; Cole, 1997; Gaskell, 1997; Magera *et al.*, 2000) and recent innovations with extended capabilities allow both small metabolite and large biomolecular analysis via LC–ESI–MS (Krishnamurthy *et al.*, 1999) and direct infusion and flow-injection ESI–MS (Goodacre *et al.*, 1999, 2000, 2002, 2003; Vaidyanathan *et al.*, 2001, 2002). In particular, LC–DAD–MS, MS–MS and MALDI–TOF may be highly automated, opening up high-throughput screening (HTS) and easier and simpler data acquisition and analysis.

Ferenci and co-workers have adopted a metabolomic approach to the analysis of *E. coli* (Liu *et al.*, 2000; Tweeddale *et al.*, 1998, 1999). They used radioisotopic labelling and extracted the cells, and apparently the medium, with a final concentration of 67% boiling ethanol for 30 min. One may assume that only the most stable metabolites survived this treatment, and indeed only about a dozen were identified via 2D thin-layer chromatography. We have chosen to use mass spectrometry for our analyses as it is a sensitive and rapid technique, does not require radio-isotopes, and is potentially capable of discriminating many more metabolites (than TLC) from their mass/charge ratio alone (and if such is available to identify many more via tandem analyses; Rashed *et al.*, 1997; Vaidyanathan *et al.*, 2002). Additionally, FT–IR, a method which measures the overall composition of a sample by detecting the molecular vibrations and other motions of chemical bonds, is another excellent method for rapid screening of microbial samples (Goodacre *et al.*, 1998a, 1998b; Naumann *et al.*, 1995; Oliver *et al.*, 1998; Timmins *et al.*, 1998).

Copyright © 2003 John Wiley & Sons, Ltd. 

**Table 1.** Genotypes of strains provided by Professor Yanofsky and used in the present work

| **W3110** | **Wild-type** | |
|---|---|---|
| CY15682 | *TrpR2* (repressor minus) | *trpR2* is a tryptophan repressor |
| CY15000 | *TnaA2* (tryptophanase minus) | Prevents tryptophan degradation |
| CY15001 | *TrpR2 tnaA2* | No repressor and no tryptophanase |
| CY15602 | Δ*trpEA2* (trp operon deleted) | Entire operon deleted |
| CY15680 | *TrpR2* Δ*trpEA2* | trp repressor and operon deleted |
| CY15681 | *TnaA2 trpA46PR9* (*trpA* bradytroph) | tnase deleted |

However, metabolomic fingerprinting of microbial strains has two major difficulties: (a) the turnover time of the intracellular metabolites can be very fast (De Koning and van Dam, 1992), necessitating the very rapid quenching of metabolism; and (b) the intracellular volume of a typical microbial suspension at a concentration of 1 mg wet weight/ml occupies only some 0.1% of the total volume, and removing the small intracellular volume from the much larger extracellular volume can (indeed, is likely to) lead to contamination of the former by the latter. However, given that microbes are rarely if ever enjoying balanced growth, and must (and do) secrete any number of substances into the medium, it occurred to us that we might make a virtue of necessity by exploiting the fact that what is secreted must reflect the exact genetic make-up of the strain in question (Allen *et al.*, 2003) and might therefore be used, according to the principles of 'guilt by association' (Oliver, 2000) or supervised learning (Kell and King, 2000), for the purposes of functional genomics in gene knockout strains. What was not known, however, was whether the metabolic footprints would be either sufficiently reproducible or discriminating as a different kind of 'fingerprinting' technique (Fiehn, 2001) to allow such discrimination in *E. coli*. We therefore decided to test this hypothesis explicitly.

We investigated tryptophan metabolism mutant strains selected by Yanofsky and co-workers (Khodursky *et al.*, 2000a; Yanofsky and Horn, 1994), who studied changes in gene expression profiles of the strains in response to tryptophan-supplemented and partially and/or totally tryptophan-starved conditions in defined media (Tao *et al.*, 1999). These strains had deletions in tryptophan repressor gene (*trpR)* or tryptophanase gene (*tnaA2*) (Kamath and Yanofsky, 1992), tryptophan operon deletion (*trpEA2*) and a leaky auxotroph, *trpA* bradytroph (*trpA46PR9*) (see Table 1). Hierarchical clustering of the profiles revealed changes in expression of a total of 691 genes with identification and functional roles assigned to 169 of the genes. As the transcriptome profiling showed specific functional alterations (Featherstone and Broadie, 2002; ter Kuile and Westerhoff, 2001) we predicted that there might be metabolite changes as well and these changes were monitored in filtered culture supernatants and sample analysis [after cellular metabolite ('fingerprinting') extraction using both perchloric and hot ethanol methods after LC separation of a range of metabolites and direct culture supernatant ('footprinting')], using FT–IR and ESI–MS. The FT–IR and MS spectral profiles were processed chemometrically (Beavis *et al.*, 2000; Goodacre and Kell, 2003; Raamsdonk *et al.*, 2001; Shaw *et al.*, 1999a; Smith, 1998).

### Materials and methods

Strains, kindly provided by Professor Yanofsky, with genotypes as shown in Table 1, were grown in defined media composed of 0.2 g glucose, 0.2 g $MgSO_4.7H_2O$, 2 g citric acid, 10 g anhydrous $K_2HPO_4$ and 3.5 g $NaNH_4HPO_4{\cdot}4H_2O$ per litre (Heatwole and Somerville, 1992), with added indole acrylate or tryptophan where stated. The inoculum was grown under the same conditions as the experimental flasks in 2 ml volumes in sterile tubes for 24 h at 30 °C and 200 rpm. Experimental 500 ml flasks with 100 ml medium were grown for 24 h, as above. The final $OD_{600}$ of the cultures was approx 2, equivalent to final cell densities of some $10^9$/ml.

### Intracellular metabolite (fingerprinting) extraction methods

Duplicate samples for metabolite extraction were withdrawn 24 h after growth.

Copyright © 2003 John Wiley & Sons, Ltd. 

### Perchloric acid extraction

A 5 ml volume of a culture was squirted into 15 ml −40 °C pre-chilled 60% methanol buffered with 70 mM HEPES/KOH, pH 7.5, mixed rapidly and centrifuged at −20 °C for 5 min at 5000 × *g*. The supernatant was discarded and the pellet was resuspended in 2 ml 35% (v/v) perchloric acid and stored at −80 °C for 1 h, thawed on ice and centrifuged as before. A 2 ml volume was withdrawn from the supernatant and neutralized with 4 × 200 µl 5 M $K_2CO_3$ (pH checked and adjusted if necessary). The sample was frozen at −80 °C, thawed and pH-monitored again. It was re-centrifuged and the supernatant was removed, aliquoted and stored frozen at −40 °C. For the control, a 5 ml volume was treated similarly to the metabolite extraction with perchloric acid (Meyer *et al*., 1999). Perchloric acid was preferred here to ethanol extraction as many more metabolites were extracted (unpublished; but see also Buchholz *et al*., 2001).

### Culture supernatant sample preparation (footprinting)

Extracellular secretion of intracellular metabolites was monitored in the culture medium after 24 h growth of the bacterial strains under varying growth conditions at 30 °C and 200 rpm, with cells removed using 0.22 µm filter units. Controls were fresh media at 0 h and wild-type grown on freshly prepared medium on three separate days with samples taken for analysis.

In this type of strategy (Hastie *et al*., 2001; Kell and King, 2000), the accepted norm is to 'train' using a subset of samples and project in the data from a different set of replicates to ensure (i.e. demonstrate) that one is not overfitting the data. Thus we used a number of different replicates from different days for this purpose (see legends to Figures).

### FT–IR analysis

Analysis by FT–IR with automated HTS was carried out with each sample run as six replicates of 10 µl volume/well on 100-well aluminium plate (Goodacre *et al*., 1998a). The plate was oven-dried at 50 °C for 30 min prior to analysis and loaded onto the motorized stage of a reflectance TLC accessory of a Bruker IFS28 FT–IR spectrometer (Bruker Spectrospin, Coventry, UK) equipped with a mercury–cadmium–telluride (MCT) detector cooled with liquid $N_2$. The spectral range was 4000–600 $cm^{-1}$ and 256 co-adds were used (Winson *et al*., 1997).

### ESI–MS analysis for fingerprinting

A Waters Alliance 2690 HPLC linked to a Photodiode array detector 996 (DAD) and Micromass LCT electrospray mass spectrometer were used for analysis of the metabolites. A 10 µl sample volume was first separated on a 200 × 4 mm chiral Nucleodex $\beta$-OH column using 12 mM ammonium acetate : methanol (99 : 1) as eluent at a flow rate of 500 µl/min and an isocratic gradient with a 10 min metabolite separation and 30 min column wash. A 40 µl/min stream was directed after splitting the volume into the MS for further analysis in the range 65–815 $m/z$. The MS was optimized with capillary voltage at 2000 V, source temperature 80 °C, desolvation temperature at 150 °C, nebulizer and desolvation gas flow at 90 and 540l/h, and sample cone and extraction cone voltage at 40 V and 11 V, respectively.

### ESI–MS for footprinting

The automated analysis was performed using the same instruments as above. Samples were diluted 10-fold in 30% HPLC grade methanol and 0.1% formic acid made up to volume with HPLC grade water. The samples were de-gassed and large particles were removed by microcentrifugation (Eppendorf microfuge) at full speed for 3–5 min. Volumes of 100 µl were dispensed into pre-labelled glass inserts and placed in tubes in the LC carousel. A 20 µl volume sample was loaded into the sample loop using LC solvents (70% 10 mM formic acid/30% HPLC grade methanol) and pumped at 0.5 ml/min. The total scan cycle was 1 s (0.9 s scan and 0.1 s interscan delay) and the complete run time was 2 min. The MS was optimized, leading to the following final conditions: capillary voltage at 2000 V, source temperature at 80 °C, desolvation temperature at 150 °C, nebulizer and desolvation gas flow at 90 and 540 l/h, and sample cone and extraction cone voltage at 40 V and 11 V, respectively (Vaidyanathan *et al*., 2001).

Copyright © 2003 John Wiley & Sons, Ltd. 

### Chemometric data processing methods

The FT–IR and mass spectrometric methods described above produce vast amounts of potentially useful data (Benton, 1996), e.g. LC–MS produces a spectrochromatogram (an array of the MS vs. time) for each sample analysed plus a diode array detector trace. Each spectrochromatogram can typically hold $10^6$ values (depending upon the MS range and sampling rates). In their native form, such data are extremely difficult to interpret. To turn such data into information of chemical or biological interest, some sort of multivariate statistical analysis must be employed.

### Data processing for FT–IR spectral analysis

Raw data were exported to MATLAB as a matrix object using the Opus software (Bruker Spectrospin). Data preprocessing was carried out on autoscaling by normalization to unit variance (Winson *et al*., 1997).

### Cluster analysis of FT–IR spectra

### Principal components analysis

PCA (Causton, 1987; Jolliffe, 1986; and see below) was performed on the original data set to give a new reduced set of orthogonal variables called principal components (PCs), the first few of which typically account for >95% of the variance.

### Discriminant function analysis

DFA is a supervised projection method (Manly, 1994); *a priori* information about sample grouping in the data set is used to produce measures of within-group variance and between-group variance. This information is then used to define discriminant functions that optimally separate the *a priori* groups (in this case the groups are defined as replicates). In this implementation, the first *n* PC scores are used as the data source for DFA, where *n* is chosen using cross-validation (Radovic *et al*., 2001).

### ESI–MS preprocessing

In order to simplify any subsequent statistical analysis, two simple pre-processing algorithms were applied to the ESI–MS spectrochromatograms. First, each ESI–MS array was reduced into a single 'aggregate' MS vector by summing the ion counts of a given $m/z$ ratio over the total scan cycle. Each MS vector was then 'binned' to unit $m/z$ ratio (i.e. ion counts of fractional $m/z$ ratios were added to the nearest integer $m/z$). Thus, after this initial data reduction an ESI–MS spectrochromatogram with MS range 65–815 $m/z$ will be reduced to a single vector having 750 values. This is a highly efficient strategy since, depending on the scan rate, the file sizes are reduced from tens of megabytes to a few kilobytes.

### Multivariate analysis

Before employing any multivariate analysis each MS vector is normalized to the total ion count (which is given a value of $10^6$). This is done so that different spectra can be compared quantitatively. Once a set of $N$ spectra (with mass range $p$) is concatenated into a single matrix ($N$ objects $\times$ $p$ variables) each column of the data set can be optionally normalized to unit variance. This is done to eliminate bias, in subsequent analysis, toward any column that contains either large absolute values or large variances (Martens and Næs, 1989). However, we note that normalization can sometimes be more detrimental than helpful. If there are a large number of redundant variables in the data, the noise on such variables is amplified to the same importance as relevant variables. This can easily cloud any underlying statistical trends.

In order to cluster the spectral data, principal components analysis (PCA) was used. (Causton, 1987; Jolliffe, 1986). PCA involves projecting the original X-matrix ($N \times p$) onto a $d$-dimensional subspace using a projection (or *loading*) matrix, thus creating object coordinates (a *score* matrix) in a new coordinate system. This is achieved by the method known as singular value decomposition (SVD) of $X$:

$$X_{N\times p} = U_{N\times d}\Lambda_{d\times d}L^T_{p\times d} = T_{N\times d}L^T_{p\times d}$$

where $U$ is the unweighted (normalized) score matrix and $T$ is the weighted (or biased) score matrix. $L$ is the loading matrix, where the columns of $L$ are known as eigenvectors or loading-PCs. $\Lambda$ is a diagonal matrix (i.e. all of the off-diagonal elements are equal to zero) containing the square roots of the first $d$ eigenvalues of the co-variance matrix ($X^TX$) where, $d < N$ and $d < p$.

Copyright © 2003 John Wiley & Sons, Ltd. 

The principal components (PCs) can be considered as a basis set used to project the original data matrix, $X$, onto the scores, $T$. In other words, the new coordinates are linear combinations of the original variables, e.g. the elements of the first principal component can be represented as:

$$t_{11} = x_{11}l_{11} + x_{12}l_{21} + \ldots + x_{1p}l_{p1}$$
$$t_{21} = x_{21}l_{11} + x_{22}l_{21} + \ldots + x_{2p}l_{p1}$$
$$\vdots$$
$$t_{n1} = x_{n1}l_{11} + x_{12}l_{n1} + \ldots + x_{np}l_{p1}$$

The influence of each of the original variables on the new PCs (i.e. the contents of the loading matrix) is determined on the basis of the maximum variance criterion. The first PC is considered to lie in the direction describing maximum variance in the original data. Each subsequent PC lies in an orthogonal direction of maximum variance that has not been considered by the former components. The number of PCs computed for a given data set is up to the analyst; however, usually as many PCs are calculated as are needed to explain a pre-set percentage of the total variance in the original data (the total number of PCs possible is equal to the number of original variables). It is also possible to use PCA analysis on a subset of the variables chosen via a genetic algorithm (GA) (Broadhurst *et al.*, 1997) and we have exploited such GA–PCA analysis of the ESI–MS data here. In particularly favourable cases the discrimination can be made on the basis of just two or three variables, which allows the display of data in a 2D or 3D plot of the actual variables themselves (as opposed to the PCs: Taylor *et al.*, 1998). Finding these variables is a combinatorial optimization problem (Cook *et al.*, 1998), as the number of pairs and triplets which can be formed from 750 (i.e. the mass spectral) variables is respectively 280 875 and 70 031 500; hence the need for the GA.

### Results and discussion

The synthesis, utilization and degradation of tryptophan in *E. coli* has been studied extensively, with regulation of its operon being effected by both repression and attenuation (transcription termination) (Yanofsky, 2000; Yanofsky and Horn, 1994; Yanofsky *et al.*, 1993, 1996). More recently a selected set of mutants and wild-type W3110 (control) strains were used to study the changes in expression profiles in response to altered tryptophan availability during early growth phase, and 15 genes organized in nine operons exhibited changes. The set of experiments conducted here for our study were different from those of Khodursky *et al.* (2000b) as the cultures were grown for 24 h into stationary phase. The wild-type W3110 and mutant strains with *trpR2* (repressor minus), *tnaA2* (tryptophanase minus), *trpEA2* (tryptophan operon minus) and *trpA bradytroph* were grown under three different growth conditions: in minimal medium; in the presence of excess tryptophan; and tryptophan starvation induced by indole acrylate (a tryptophan analogue). Indole acrylate prevents tryptophan repression (Ilic *et al.*, 1999; Isaacs *et al.*, 1994) by inhibiting the charging of tRNAtrp by tryptophanyl-tRNA synthetase, which in turn effects both repression and attenuation (transcription termination) of the tryptophan operon. As arginine biosynthetic genes are sensitive to tryptophan starvation, very mild starvation conditions were imposed and ideally a study would involve the use of near-isogenic strains (Khodursky *et al.*, 2000b). Intracellular metabolite data are not displayed in this report as no meaningful clustering of replicates was observed. However, preliminary experimental results had indicated that differences between strains could be detected using the filter-sterilized media samples from cultures grown for 24 h, and therefore these samples were analysed using FT–IR and mass spectrometry.

### FT–IR analysis of *E. coli* tryptophan metabolism mutants

FT–IR spectral analysis is used routinely in our laboratory for high-throughput screening of a wide range of microbes (Goodacre *et al.*, 1998b; Oliver *et al.*, 1998) and their products (McGovern *et al.*, 1999, 2002; Shaw *et al.*, 1999b; Winson *et al.*, 1997, 1998). The data produced by FT–IR spectroscopy are multidimensional and thus chemometric data analysis is required. Additionally, characteristic vibrations can lead to the identification of specific metabolites (e.g. Goodacre *et al.*, 2000; Johnson *et al.*, 2000; McGovern *et al.*, 2002).

The DFA biplots of FT–IR data from replicate samples are shown in Figures 1, 2 and 3, with representative FT–IR spectra being illustrated in Figure 1A. Figure 1 shows *E. coli* W3110 wild-type strain grown for 24 h in normal growth

Copyright © 2003 John Wiley & Sons, Ltd. 

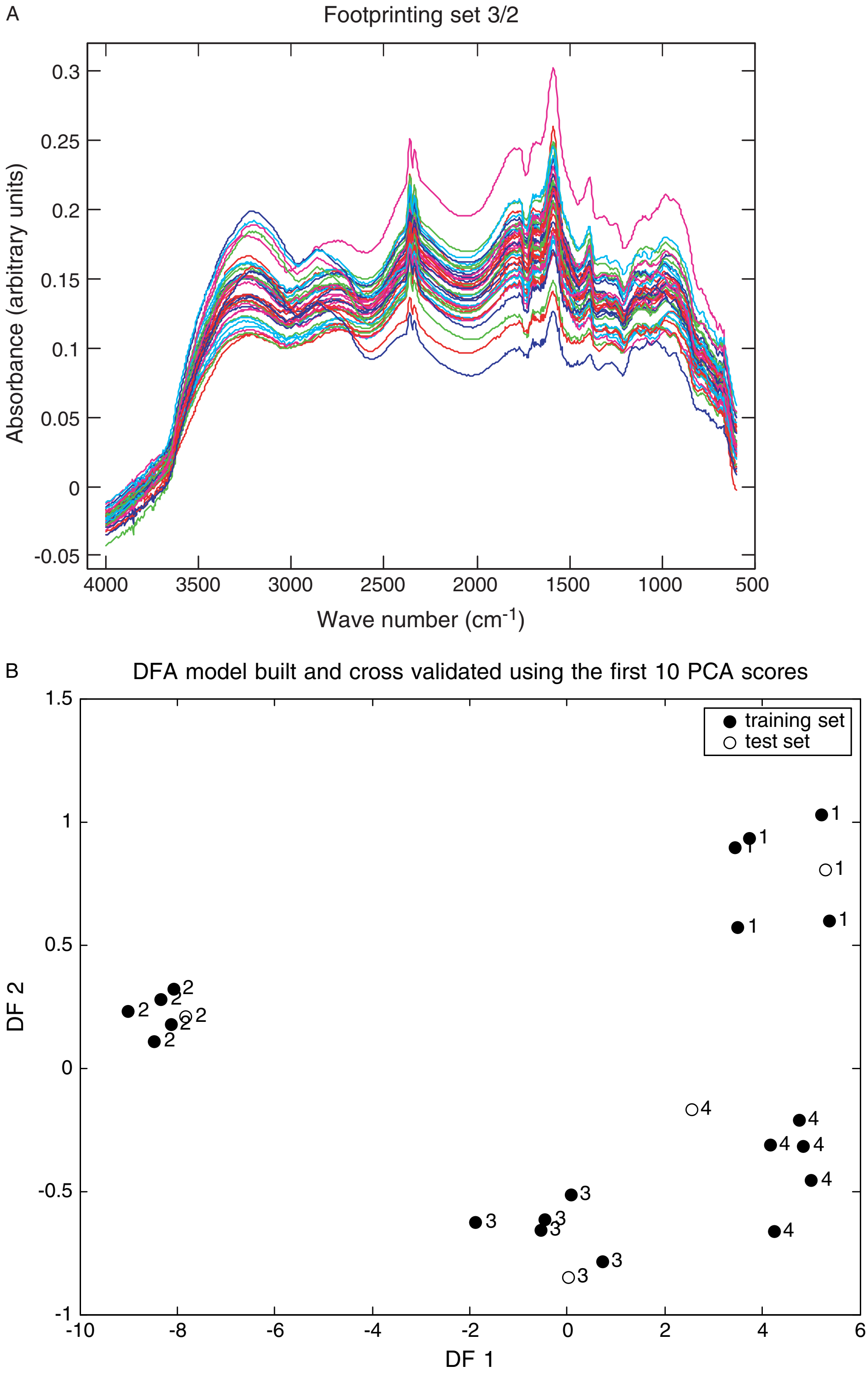

A

Footprinting set 3/2

Absorbance (arbitrary units)

0.3

0.25

0.2

0.15

0.1

0.05

0

-0.05

4000

3500

3000

2500

2000

1500

1000

500

Wave number (cm-1)

B

DFA model built and cross validated using the first 10 PCA scores training set test set

DF 2

1.5

1

0.5

0

-0.5

-1

-10

-8

-6

-4

-2

0

2

4

6

DF 1

medium without any additions (1), in medium supplemented with 50 µg/ml tryptophan (2) or indole acrylate at 10 µg/ml (3) and 15 µg/ml (4). Clustering of the five replicates for each of the samples is well defined. That the projected spectra are recovered in the correct group clearly demonstrates the high reproducibility of FT–IR here. The samples in the presence of added tryptophan (2) and/or indole acrylate (3 and 4) are clearly separating away from the normal minimal growth medium (1) and tryptophan samples cluster away from the indole acrylate samples.

Figure 2 shows the DFA biplot of selected tryptophan mutant strains of *E. coli* grown in minimal medium only. The replicates and projected data cluster together for each sample and W3110 (1) clusters away from the other strains. The tryptophanase-negative strains, *tnaA2* (2) and *tnaA2* bradytroph (3) cluster together, whereas *trpR2 mutant* (4) harbouring a repressor deletion clusters away from the other strains. The DFA of selected tryptophan mutant strains grown in the presence of added tryptophan in Figure 3 show that the tryptophan repressor, *trpR2,* deletion strain and

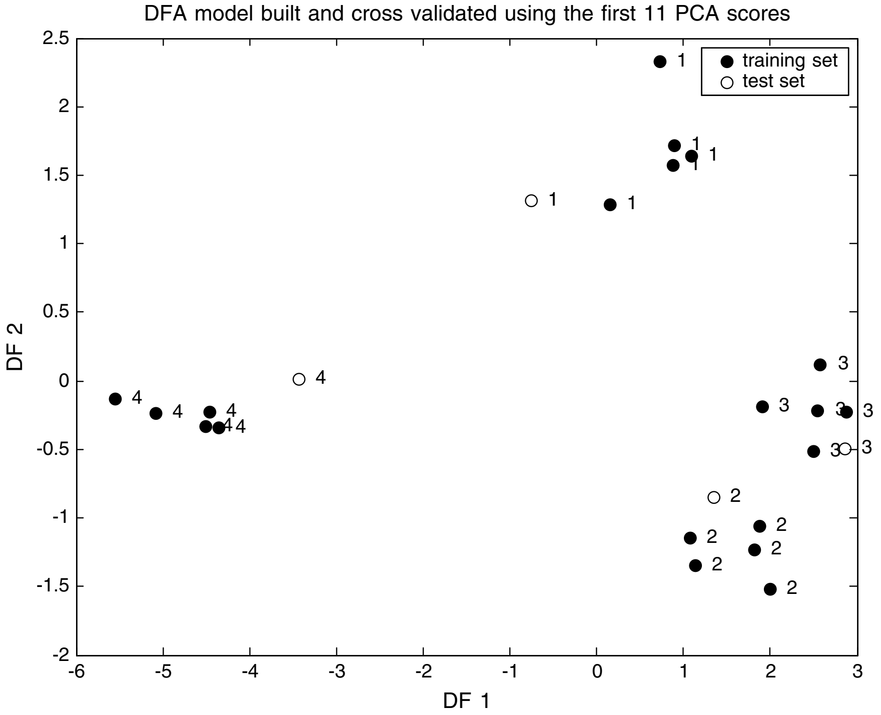

**Figure 2.** DFA biplot of FT–IR data showing the relationship between culture media from wild-type W3110 (1) 15000 *tnaA2* (2), 15681 *tnaA2 trpA46PR9* bradytroph (3) and 15682 *trpR2* (4) strains grown in minimal media only. Cross-validation of the DFA model was performed, whereby the original data set was divided into two subsets, one of which was used to train the model (closed circle) and the other subsequently used to validate it (open circle). This process serves to ensure that the optimal number of principal components (PCs) is used to build the DFA model; in this case, 11 PCs were needed

**Figure 1.** (A) Illustrative FT–IR data from stationary phase supernatants of wild-type and mutants of *E. coli*, prepared as described in the text. (B) DFA biplot of FT–IR data showing the relationship between 24 h culture media of wild-type W3110 grown in minimal medium only (1), in minimal medium in the presence of 50 µg/ml tryptophan (2), and with 10 µg/ml (3) and 15 µg/ml (4) indole acrylate (induces tryptophan starvation). Cross-validation of the DFA model was performed, whereby the original data set was divided into two subsets, one of which was used to train the model (closed circle) and the other subsequently used to validate it (open circle). This process serves to ensure that the optimal number of principal components (PCs) are used to build the DFA model and that the clustering relationships in the data subsequently observed are real, and not an artefact of, for example, over-fitting (i.e. to fit some of the random variation in the data as if it were deterministic structure), which tends to arise when too many principal components are employed. In this case the optimal number of PCs was 10

Copyright © 2003 John Wiley & Sons, Ltd. 

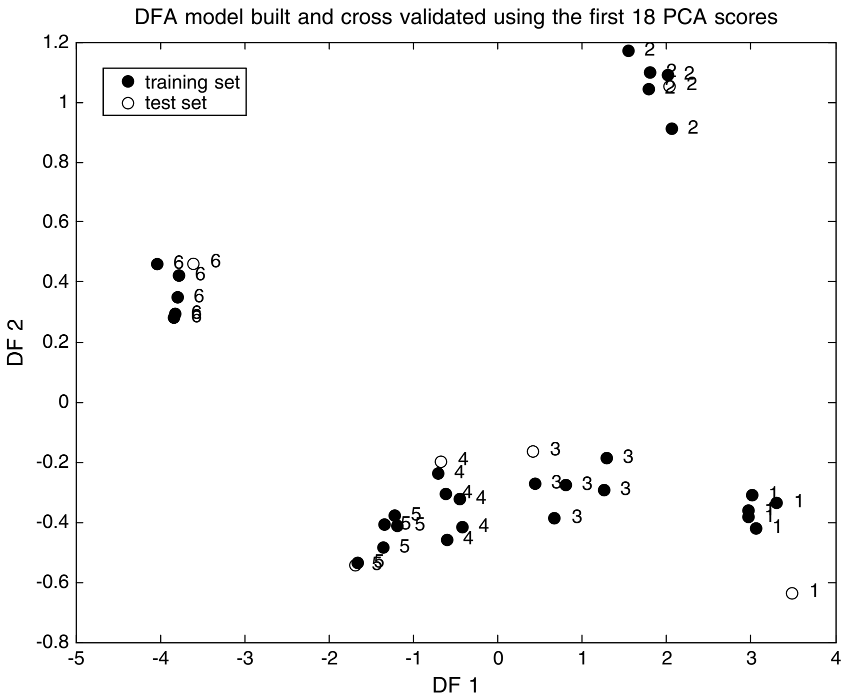

**Figure 3.** DFA biplot of FT–IR data showing the relationship between culture media from wild-type W3110 (1), 15682 *trpR2* (2), 15000 *tnaA2* (3), 15001 *trpR2 tnaA2* (4), 15602 Δ*trpEA2* (5) and 15680 *trpR2* Δ*trpEA2*(6) strains grown in minimal media supplemented with 50 μg/ml tryptophan (which causes tryptophan repression). Cross-validation of the DFA model was performed, whereby the original data set was divided into two subsets, one of which was used to train the model (closed circles) and the other subsequently used to validate it (open circles). This process serves to ensure that the optimal number of principal components (PCs) is used to build the DFA model; in this case, 18 PCs were needed total tryptophan operon deletion (6) cluster away from the other strains and the W3110 wild-type but are closer to the strain with the operon deletion only (5). Similarly, the strain with only *trpR2* deletion (2) clusters away from all the other strains but is closer to *tnaA2* and *trpA2* deletions (4) and *tnaA2* deletion (3) strains.

The distinct pattern of clustering of media using FT–IR data analysis derived from a single strain cultured under diverse growth conditions clearly suggested that there are obvious changes in the extracellular metabolite composition. This could be induced by growth media supplemented with tryptophan or indole acrylate, because tryptophan metabolism is tightly regulated by the presence of tryptophan and indole acrylate in the medium. These changes could also be attributed to the uptake of nutrients from the growth medium or secretion of intracellular metabolites into the medium during growth. Using a subset of strains carrying defined gene mutations, DFA analysis shows distinct clusters, which are wholly reproducible at the mutant level, as confirmed by the projection of 'unknown' biological replicates into PC-DFA space. Strains with single gene deletions for tryptophanase and/or tryptophan repressor proteins show DFA clusters displaced from those of the strains carrying the polycistronic deletion of tryptophan operon. Thus a marked effect on tryptophan metabolism is generated by single or multiple gene deletions. In conclusion, clustering using FT–IR analysis can easily separate strains according to their genotype and thus metabolomics can provide a rapid high-content screen for genetic lesions.

### ESI–MS analysis of *E. coli* tryptophan metabolism mutants

Additionally, these samples were also analysed using direct injection mass spectrometry, which has been used successfully to identify bacteria

Copyright © 2003 John Wiley & Sons, Ltd.

from crude cell-free extract preparations via a complex milieu of large and small chemicals (Magera *et al.*, 2000; Morris and Cooper, 2000; Tiller *et al.*, 2000; Vaidyanathan *et al.*, 2001, 2002; van Eijk *et al.*, 1999). Samples of 24 h culture media for this study were also analysed using ESI–MS in the positive ion mode, as we were focusing on changes in tryptophan metabolites. Mass spectrometric data are also high-dimensional and must be preprocessed before chemometric analysis. Representative ESI–MS spectra are given in Figure 4A.

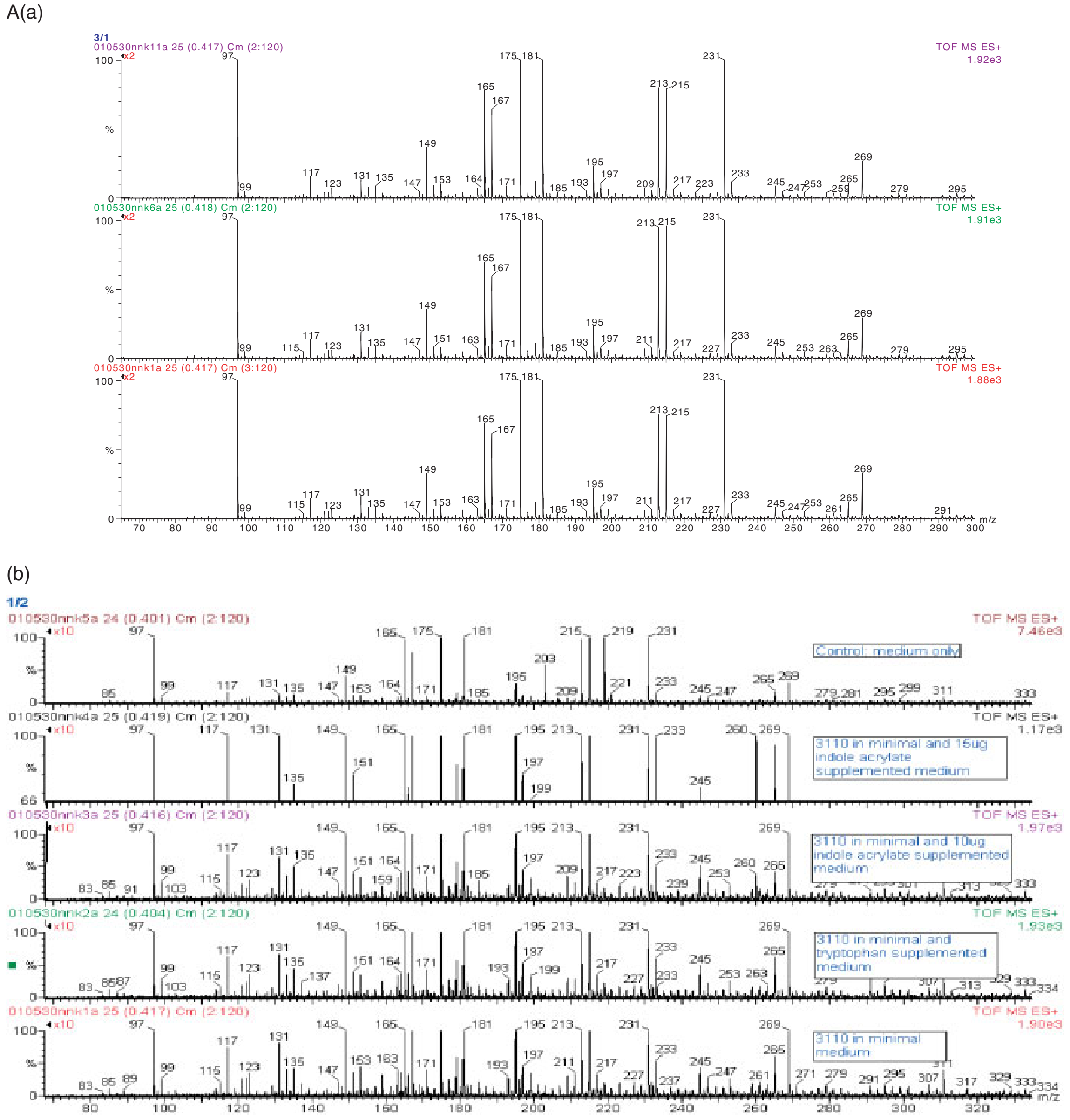

**Figure 4.** (A) Illustrative mass spectral data from stationary phase supernatants of wild-type strains of *E. coli* prepared as described in the text, (a) three separate experiments to show reproducibility and (b) in different media. (B) GA–PCA-derived plot of ESI–MS data of culture media from wild-type W3110 grown in the presence of 50 μg/ml tryptophan (1), 10 μg/ml (2) and 15 μg/ml (3) indole acrylate for 24 h. The MS data of wild-type W3110 grown in minimal medium alone were subtracted as background masses. The axes represent the normalized ion counts of the stated *m*/*z* variables

Copyright © 2003 John Wiley & Sons, Ltd.

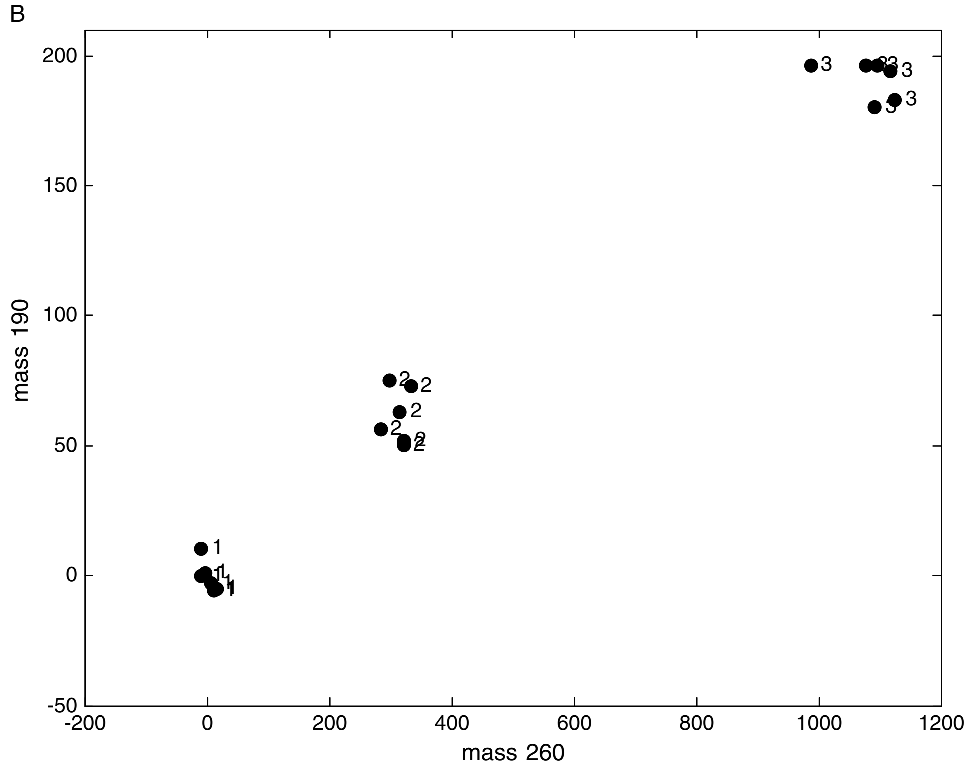

**Figure 4.** Continued

The GA–PCA-derived plots of ESI–MS showing selected *m/z* ions for filter-sterilized culture media of wild-type W3110 and the other strains grown for 24 h are shown in Figures 4, 5 and 6. All plots show very close clustering of the six replicate samples. In order to remove the effect of the wild-type's growth on the metabolic footprints, the MS data of the wild-type, *E. coli* W3110, grown only in minimal medium, were subtracted as a 'background' from all the sample data shown in these figures.

The analysis showed that the samples of wild-type grown in different media in Figure 4 could be clearly discriminated using just two analyte ions. The *m/z* 190 and 260, alone or together, allowed clear separation of the wild-type grown in supplemented medium with indole acrylate at 15 µg/ml (2), showing the greatest variance with *m/z* 260 at around 1150 normalized ion counts (NIC) and with *m/z* 190 at around 170 NIC. In the presence of indole acrylate at the lower concentration of 10 µg/ml (1), there were around 360 NIC of *m/z* 260 and around 75 NIC for *m/z* of 190. By contrast, medium supplemented with tryptophan (0) showed no significant discrimination using either of these masses and clustered around zero at the origin.

Figure 5 shows a 2D *m/z* plot of 260 vs. 381 derived from GA–PCA of the strains grown in minimal media only. The *trpR2* (7) strain clearly separated from the others with *m/z* of 381 and about 250 NIC and *tnaA2 trpA46PR9* bradytroph (6) separated with *m/z* 260 only with an NIC of around 450. The *tnaA2* (5) strain clustered around zero, suggesting that it was similar (at least in these two analytes) to the wild-type.

The GA–PCA-derived plot of the organisms grown in the presence of 50 µg/ml tryptophan is shown in Figure 6. This pseudo-3D plot of *m/z* 115 243 and 288 clearly distinguished the *trpR2* (10), *tnaA2* (11), *trpR2 tnaA2* (12), Δ*trpEA2* (13) and *trpR2* Δ*trpEA2* (14) into five tight clusters. The degradation of tryptophan leads to indole (Goodacre and Kell, 1993; Prinsen *et al.*, 1997), pyruvate and ammonia, and the MS analysis of the supernatant medium shows that a mass of 288 clearly discriminates *trpR2 tnaA2* and Δ*trpEA2*

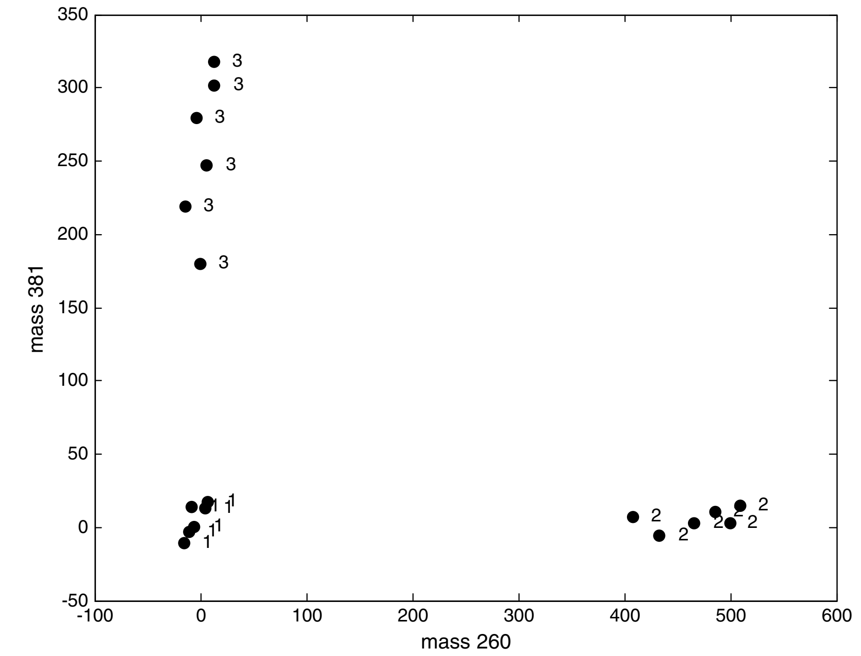

**Figure 5.** GA–PCA-derived plot of ESI–MS data of culture media from 15000 *tnaA2* (1), 15681 *tnaA2 trpA46PR9* bradytroph (2) and 15682 *trpR2*(3) strains grown in minimal media only for 24 h. The MS data of wild-type W3110 were subtracted as background masses from the MS data of each of the mutant strains. The axes represent the normalized ion counts of the stated *m*/z variables

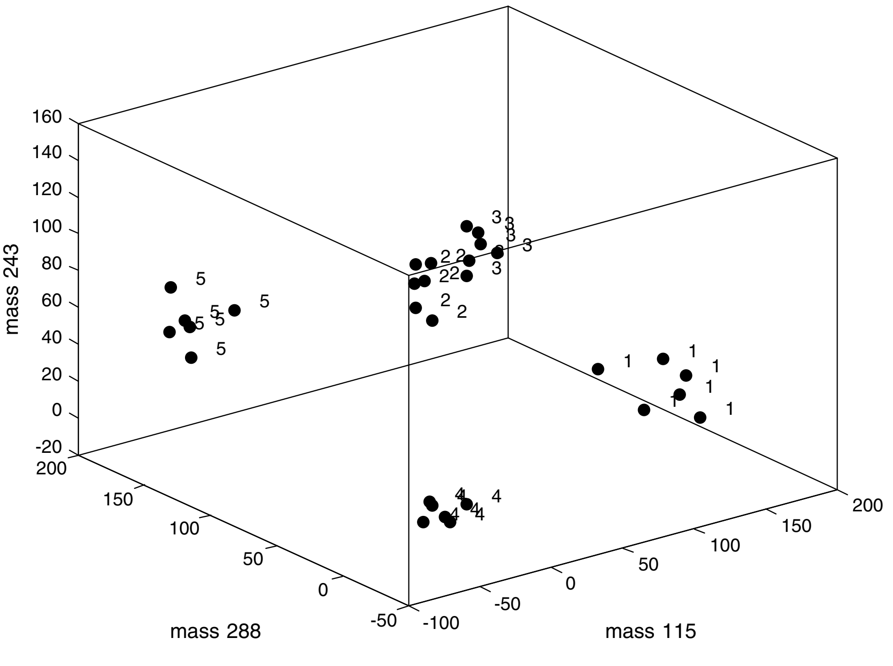

**Figure 6.** Pseudo-3D GA–PCA-derived plot of ESI–MS data of culture media from 15682 *trpR2* (1), 15000 *tnaA2* (2), 15001 *trpR2 tnaA2* (3), 15602 Δ*trpEA2* (4) and 15680 *trpR2* Δ*trpEA2*(5) strains grown in minimal media supplemented with 50 μg/ml tryptophan for 24 h. The MS data of wild-type W3110 were subtracted as background masses from the MS data of each of the mutant strains. The axes represent the normalized ion counts of the stated *m*/z variables

Copyright © 2003 John Wiley & Sons, Ltd. 

strains. Indole-3-glycerol phosphate is the penultimate intermediate of tryptophan synthesis, and has a mass of 287, which, when protonated in positive-ion ESI–MS, gives it an *m/z* of 288 (Mohammed *et al.*, 1999). It is thus highly likely that the *m/z* 288 analyte is therefore indole-3-glycerol phosphate, and a functional genomics strategy with access to a tandem instrument would establish this.

In conclusion, these rapid spectroscopic methods allowed us to discriminate these closely related single-gene knockout strains from their metabolic footprints alone. Thus, they can be used to detect small phenotypic differences that other conventional phenotyping and global profiling approaches would miss, opening up the possibility of gaining useful information from knockouts with subtle phenotypes, especially in functional genomics studies with large libraries of such gene knockouts. The footprinting approach, which does not *rely* upon the identification of any peaks, can be used without prior knowledge of the likely function of the genes of interest, and can supply data that could indicate potential functions for genes. The FT–IR is rapid but is chemically unselective, and is better for a very rapid 'fingerprinting' type of study in which it is not of great interest to identify the metabolites of interest (Fiehn, 2001). By contrast, the ESI–MS is slightly slower but can give an indication of the metabolites contributing to the differences between the strains. More definitive identification would require other methods, such as tandem mass spectrometry. Nevertheless, as rapid and reagentless approaches, FT–IR and ESI–MS of metabolic footprints are both much quicker and cheaper than are transcriptomics and proteomics.

### Acknowledgements

We thank the BBSRC for financial support, and Simon Andrews, Arne Buchholz, Simon Doig and Sue Stovell for useful discussions.

### References

Abel CBL, Lindon JC, Noble D, *et al.* 1999. Characterization of metabolites in intact *Streptomyces citricolor* culture supernatants using high-resolution nuclear magnetic resonance and directly coupled high-pressure liquid chromatography–nuclear magnetic resonance spectroscopy. *Anal Biochem* **270**: 220–230.

Akashi H, Gojobori T. 2002. Metabolic efficiency and amino acid composition in the proteomes of *Escherichia coli* and *Bacillus subtilis*. *Proc Natl Acad Sci USA* **99**: 3695–3700.

Allen JK, Davey HM, Broadhurst D, *et al.* 2003. Metabolic footprinting: a high-throughput, high-information approach to cellular characterisation and functional genomics. *Nature Biotechnol* **21**: 692–696.

Auer G, Alaiya A, Bergman AC, Bergman T. 1998. From genome to proteome: multiple gene expression analysis in human tumors. *Cytology: Abstracts; XIIIth International Congress of Cytology*, Tokyo, Japan, May 10–14, 1998; 460.

Beavis RB, Colby SM, Goodacre R, *et al.* 2000. Artificial intelligence and expert systems in mass spectrometry. In *Encyclopedia of Analytical Chemistry*, Meyers RA (ed.). Wiley: Chichester; 11 558–11 597.

Benton D. 1996. Bioinformatics — principles and potential of a new multidisciplinary tool. *Trends Biotechnol* **14**: 261–272.

Blattner FR, Plunket G, Perna N, *et al.* 1998. Comparative genome sequencing of *E. coli* O157:H7 vs. *E. coli* K-12. In *Proceedings of the 1998 Miami Bio/Technology Winter Symposium*, Ahmad F, Baumbach L, Bernstein P, *et al.* (eds). IRL Press: Oxford; 3–4.

Blattner FR, Plunkett G, Bloch A, *et al.* 1997. The complete genome sequence of *Escherichia coli* K-12. *Science* **277**: 1453–1474.

Brent R. 1999. Functional genomics: learning to think about gene expression data. *Curr Biol* **9**: R338–341.

Brent R. 2000. Genomic biology. *Cell* **100**: 169–183.

Broadhurst D, Goodacre R, Jones A, Rowland JJ, Kell DB. 1997. Genetic algorithms as a method for variable selection in multiple linear regression and partial least squares regression, with applications to pyrolysis mass spectrometry. *Anal Chim Acta* **348**: 71–86.

Buchholz A, Takors R, Wandrey C. 2001. Quantification of intracellular metabolites in *Escherichia coli* K12 using liquid chromatographic–electrospray ionization tandem mass spectrometric techniques. *Anal Biochem* **295**: 129–137.

Burge CB. 2001. Chipping away at the transcriptome. *Nature Genet* **27**: 232–233.

Caceres A, Cardenas S, Gallego M, Rodriguez A, Valcarcel M. 2000. Automated flow system on-line to LC with post-column derivatization for determination of sugars in carbohydrate-rich foods. *Chromatographia* **52**: 314–318.

Causton DR. 1987. *A Biologist's Advanced Mathematics*. Allen and Unwin: London.

Champion KM, Nishihara JC, Joly JC, Arnott D. 2001. Similarity of the *Escherichia coli* proteome upon completion of different biopharmaceutical fermentation processes. *Proteomics* **1**: 1133–1148.

Choe LH, Chen W, Lee KH. 1998. Proteome analysis of factor for inversion stimulation (Fis) overproduction in *Escherichia coli*. In *Two-dimensional Electrophoresis: From Genome to Proteome*. Wiley-VCH: Siena, Italy; 798–805.

Clare A, King RD. 2002. Machine learning of functional class from phenotype data. *Bioinformatics* **18**: 160–166.

Cole RB (ed.). 1997. *Electrospray Ionization Mass Spectrometry: Fundamentals, Instrumentation and Applications*. Wiley: New York.

Cook WJ, Cunningham WH, Pulleyblank WR, Schrijver A. 1998. *Combinatorial Optimization*. Wiley: New York.

Cornish-Bowden A, Cardenas ML. 2000. From genome to cellular phenotype — a role for metabolic flux analysis? *Nature Biotechnol* **18**: 267–269.

Copyright © 2003 John Wiley & Sons, Ltd. 

Covert MW, Schilling CH, Famili I, *et al*. 2001. Metabolic modeling of microbial strains *in silico*. *Trends Biochem Sci* **26**: 179–186.

De Koning W, van Dam K. 1992. A method for the determination of changes of glycolytic metabolites in yeast on a subsecond time scale using extraction at neutral pH. *Anal Biochem* **204**: 118–123.

Devaux F, Marc P, Jacq C. 2001. Transcriptomes, transcription activators and microarrays. *FEBS Lett* **498**: 140–144.

Dunn M. 1998. Proteome analysis: chemists are analysing the total protein content of organisms to gain insights into complex cellular processes and accelerate the drug discovery process. *Chem Br* **34**: 54–58.

Featherstone DE, Broadie K. 2002. Wrestling with pleiotropy: genomic and topological analysis of the yeast gene expression network. *Bioessays* **24**: 267–274.

Fiehn O. 2001. Combining genomics, metabolome analysis, and biochemical modelling to understand metabolic networks. *Comp Funct Genom* **2**: 155–168.

Fiehn O. 2002. Metabolomics: the link between genotypes and phenotypes. *Plant Mol Biol* **48**: 155–171.

Fiehn O, Kloska S, Altmann T. 2001. Integrated studies on plant biology using multiparallel techniques. *Curr Opin Biotechnol* **12**: 82–86.

Fiehn O, Kopka J, Dormann P, *et al*. 2000. Metabolite profiling for plant functional genomics. *Nature Biotechnol* **18**: 1157–1161.

Futcher B, Latter GI, Monardo P, McLaughlin CS, Garrels JI. 1999. A sampling of the yeast proteome. *Mol Cell Biol* **19**: 7357–7368.

Futcher B, Latter J, Monardo P, McLaughlin C. 1997. The *S. cerevisiae* proteome. In *Yeast Cell Biology*. Cold Spring Harbor Laboratory Press: New York; 78.

Gaskell SJ. 1997. Electrospray: principles and practice. *J Mass Spectrom* **32**: 677–688.

Goodacre R, Heald JK, Kell DB. 1999. Characterization of intact microorganisms using electrospray ionization mass spectrometry. *FEMS Microbiol Lett* **176**: 17–24.

Goodacre R, Kell DB. 1993. Rapid and quantitative analysis of bioprocesses using pyrolysis mass spectrometry and neural networks: application to indole production. *Anal Chim Acta* **279**: 17.

Goodacre R, Kell DB. 2003. Evolutionary computation for the interpretation of metabolome data. In *Metabolic Profiling: Its Role in Biomarker Discovery and Gene Function Analysis*, Harrigan GG, Goodacre R (eds). Kluwer Academic: Dordrecht; 239–256.

Goodacre R, Rooney PJ, Kell DB. 1998a. Rapid analysis of microbial systems using vibrational spectroscopy and supervised learning methods: application to the discrimination between methicillin-resistant and methicillin-susceptible *Staphylococcus aureus*. Proceedings of SPIE: Infrared Spectroscopy: New Tool in Medicine, San Jose, CA; 220–229.

Goodacre R, Shann B, Gilbert RJ, *et al*. 2000. Detection of the dipicolinic acid biomarker in *Bacillus* spores using Curie-point pyrolysis mass spectrometry and Fourier transform infrared spectroscopy. *Anal Chem* **72**: 119–127.

Goodacre R, Timmins ÉM, Burton R, *et al*. 1998b. Rapid identification of urinary tract infection bacteria using hyperspectral whole-organism fingerprinting and artificial neural networks. *Microbiology UK* **144**: 1157–1170.

Goodacre R, Vaidyanathan S, Bianchi G, Kell DB. 2002. Metabolic profiling using direct infusion electrospray ionization mass spectrometry for the characterization of olive oils. *Analyst* **127**: 1457–1462.

Goodacre R, York EV, Heald JK, Scott IM. 2003. Chemometric discrimination of unfractionated plant extracts profiled by flow-injection electrospray mass spectrometry. *Phytochemistry* **62**: 859–863.

Goryanin I, Hodgman TC, Selkov E. 1999. Mathematical simulation and analysis of cellular metabolism and regulation. *Bioinformatics* **15**: 749–758.

Hall R, Beale M, Fiehn O, Hardy N, Sumner L, Bino R. 2002. Plant metabolomics: the missing link in functional genomics strategies. *Plant Cell* **14**: 1437–1440.

Han MJ, Yoon SS, Lee SY. 2001. Proteome analysis of metabolically engineered *Escherichia coli* producing poly(3-hydroxybutyrate). *J Bacteriol* **183**: 301–308.

Hartbrich A, Schmitz G, Weuster-Botz D, De Graaf AA, Wandrey C. 1996. Development and application of a membrane cyclone reactor for *in vivo* NMR spectroscopy with high microbial cell densities. *Biotechnol Bioeng* **51**: 624–635.

Hastie T, Tibshirani R, Friedman J. 2001. *The Elements of Statistical Learning: Data Mining, Inference and Prediction*. Springer-Verlag: Berlin.

Heatwole VM, Somerville RL. 1992. Synergism between the *trp* repressor and *tyr* repressor in repression of the *arol* promoter of *Escherichia coli*. *J Bacteriol* **174**: 331–335.

Hieter P, Boguski M. 1997. Functional genomics: it's all how you read it. *Science* **278**: 601–602.

Ilic N, Oestin A, Cohen JD. 1999. Differential inhibition of indole-3-acetic acid and tryptophan biosynthesis by indole analogues. I. Tryptophan-dependent IAA biosynthesis. *Plant Growth Reg* **27**: 57–62.

Isaacs H, Chao D, Yanofsky C, Saier MH. 1994. Mechanism of catabolite repression of tryptophanase synthesis in *Escherichia coli*. *Microbiology — Reading* **140**: 2125–2134.

Johnson HE, Gilbert RJ, Winson MK, *et al*. 2000. Explanatory analysis of the metabolome using genetic programming of simple, interpretable rules. *Genetic Progr Evolvable Machines* **1**: 243–258.

Jolliffe IT. 1986. *Principal Component Analysis*. Springer-Verlag: Berlin.

Joubert-Caron R, Caron M. 1999. Proteome and proteomics: new concepts for new fields of application in biomedicine. *Med Sci* **15**: 701–705.

Jurgen B, Hanschke R, Sarvas M, Hecker M, Schweder T. 2001. Proteome and transcriptome based analysis of *Bacillus subtilis* cells overproducing an insoluble heterologous protein. *Appl Microbiol Biotechnol* **55**: 326–332.

Kabir MM, Shimizu K. 2001. Proteome analysis of a temperature-inducible recombinant *Escherichia coli* for poly-$\beta$-hydroxybutyrate production. *J Biosci Bioeng* **92**: 277–284.

Kamath AV, Yanofsky C. 1992. Characterization of the tryptophanase operon of *Proteus vulgaris*. Cloning, nucleotide sequence, amino acid homology, and *in vitro* synthesis of the leader peptide and regulatory analysis. *J Biol Chem* **267**: 19 978–19 985.

Kanehisa M, Goto S. 2000. KEGG: *Kyoto Encyclopedia of Genes and Genomes*. *Nucleic Acids Res* **28**: 27–30.

Copyright © 2003 John Wiley & Sons, Ltd. 

Karp PD, Riley M, Paley SM, Pelligrini-Toole A. 1996. EcoCyc: an encyclopedia of *Escherichia coli* genes and metabolism. *Nucleic Acids Res* **24**: 32–39.

Karp PD, Riley M, Saier M, *et al.* 2002a. The EcoCyc database. *Nucleic Acids Res* **30**: 56–58.

Karp PD, Riley M, Saier M, *et al.* 2000b. The EcoCyc and MetaCyc databases. *Nucleic Acids Res* **28**: 56–59.

Kell DB, King RD. 2000. On the optimization of classes for the assignment of unidentified reading frames in functional genomics programmes: the need for machine learning. *Trends Biotechnol* **18**: 93–98.

Kell DB, Mendes P. 2000. Snapshots of systems: metabolic control analysis and biotechnology in the post-genomic era. In *Technological and Medical Implications of Metabolic Control Analysis*, Cornish-Bowden A, Cárdenas ML (eds). Kluwer Academic: Dordrecht; 3–25 (and see **http://qbab.aber.ac.uk/dbk/mca99.htm**).

Khodursky AB, Peter BJ, Cozzarelli NR, *et al.* 2000a. DNA microarray analysis of gene expression in response to physiological and genetic changes that affect tryptophan metabolism in *Escherichia coli*. Colloquium on Auditory Neuroscience: Development, Transduction and Integration, Irvine, CA; 12 170–12 175.

Khodursky AB, Peter BJ, Cozzarelli NR, *et al.* 2000b. DNA microarray analysis of gene expression in response to physiological and genetic changes that affect tryptophan metabolism in *Escherichia coli*. *Proc Natl Acad Sci USA* **97**: 12 170–12 175.

King RD, Karwath A, Clare A, Dehaspe L. 2000. Accurate prediction of protein functional class from sequence in the *Mycobacterium tuberculosis* and *Escherichia coli* genomes using data mining. *Yeast* **17**: 283–293.

Kose F, Weckwerth W, Linke T, Fiehn O. 2001. Visualizing plant metabolomic correlation networks using clique-metabolite matrices. *Bioinformatics* **17**: 1198–1208.

Kramer R. 1994. Systems and mechanisms of amino acid uptake and excretion in prokaryotes. *Arch Microbiol* **162**: 1–13.

Krishnamurthy T, Davis MT, Stahl DC, Lee TD. 1999. Liquid chromatography microspray mass spectrometry for bacterial investigations. *Rapid Commun Mass Spectrom* **13**: 39–49.

Liang P, Labedan B, Riley M. 2002. Physiological genomics of *Escherichia coli* protein families. *Physiol Genom* **9**: 15–26.

Liu XQ, Ng C, Ferenci T. 2000. Global adaptations resulting from high population densities in *Escherichia coli* cultures. *J Bacteriol* **182**: 4158–4164.

Loo RRO, Cavalcoli JD, VanBogelen RA, *et al.* 2001. Virtual 2D gel electrophoresis: visualization and analysis of the *E. coli* proteome by mass spectrometry. *Anal Chem* **73**: 4063–4070.

Magera MJ, Matern D, Rinaldo P. 2000. A method for the quantitative determination of methylmalonic acid in plasma by LC–MS/MS stable isotope dilution analysis. In *Inborn Errors of Metabolism*. Kluwer Academic Press: Cambridge; 96.

Manly BFJ. 1994. *Multivariate Statistical Methods: A Primer*. Chapman and Hall: London.

Martens H, Næs T. 1989. *Multivariate Calibration*. Wiley: New York.

McGovern AC, Broadhurst D, Taylor J, *et al.* 2002. Monitoring of complex industrial bioprocesses for metabolite concentrations using modern spectroscopies and machine learning: application to gibberellic acid production. *Biotechnol Bioeng* **78**: 527–538.

McGovern AC, Ernill R, Kara BV, Kell DB, Goodacre R. 1999. Rapid analysis of the expression of heterologous proteins in *Escherichia coli* using pyrolysis mass spectrometry and Fourier transform infrared spectroscopy with chemometrics: application to $\alpha$2-interferon production. *J Biotechnol* **72**: 157–167.

Mendes P. 2002. Emerging bioinformatics for the metabolome. *Brief Bioinform* **3**: 134–145.

Meyer S, Noisommit Rizzi N, Reuss M, Neubauer P. 1999. Optimized analysis of intracellular adenosine and guanosine phosphates in *Escherichia coli*. *Anal Biochem* **271**: 43–52.

Michal G (ed.) 1999. *Biochemical Pathways: An Atlas of Biochemistry and Molecular Biology*. Wiley: New York.

Mohammed N, Onodera R, Khan RI. 1999. Tryptophan biosynthesis and production of other related compounds from indolepyruvic acid by mixed ruminal bacteria, protozoa, and their mixture *in vitro*. *J Gen Appl Microbiol Tokyo* **45**: 143–148.

Morris M, Cooper D. 2000. Rapid analysis of amino acid isomers by LC–MS/MS. In *Inborn Errors of Metabolism*. Kluwer Academic Press: Cambridge; 273.

Naumann D, Keller S, Helm D, Schultz C, Schrader B. 1995. FT–IR spectroscopy and FT–Raman spectroscopy are powerful analytical tools for the non-invasive characterization of intact microbial cells. *J Mol Struct* **347**: 399–405.

Nicholson JK, Lindon JC, Holmes E. 1999. 'Metabonomics': understanding the metabolic responses of living systems to pathophysiological stimuli via multivariate statistical analysis of biological NMR spectroscopic data. *Xenobiotica* **29**: 1181–1189.

Oliver SG. 1996. From DNA sequence to biological function. *Nature* **379**: 597–600.

Oliver SG. 1997. Yeast as a navigational aid in genome analysis. *Microbiol UK* **143**: 1483–1487.

Oliver SG. 2000. Proteomics: guilt-by-association goes global. *Nature* **403**: 601–603.

Oliver SG, Winson MK, Kell DB, Baganz F. 1998. Systematic functional analysis of the yeast genome. *Trends Biotechnol* **16**: 373–378.

Oshima T, Aiba H, Masuda Y, *et al.* 2002. Transcriptome analysis of all two-component regulatory system mutants of *Escherichia coli* K-12. *Mol Microbiol* **46**: 281–291.

Ouzounis CA, Karp PD. 2000. Global properties of the metabolic map of *Escherichia coli*. *Genome Res* **10**: 568–576.

Prinsen E, Van Dongen W, Esmans EL, Van Onckelen HA. 1997. HPLC linked electrospray tandem mass spectrometry: a rapid and reliable method to analyse indole-3-acetic acid metabolism in bacteria. *J Mass Spectrom* **32**: 12–22.

Raamsdonk LM, Teusink B, Broadhurst D, *et al.* 2001. A functional genomics strategy that uses metabolome data to reveal the phenotype of silent mutations. *Nat Biotechnol* **19**: 45–50.

Radovic BS, Goodacre R, Anklam E. 2001. Contribution of pyrolysis mass spectrometry (Py-MS) to authenticity testing of honey. *J Anal Appl Pyrol* **60**: 79–87.

Rashed MS, Bucknall MP, Little D, *et al.* 1997. Screening blood spots for inborn errors of metabolism by electrospray tandem mass spectrometry with a microplate batch process and a computer algorithm for automated flagging of abnormal profiles. *Clin Chem* **43**: 1129–1141.

Riley M, Serres MH. 2000. Interim report on genomics of *Escherichia coli*. *Ann Rev Microbiol* **54**: 341–411.

Copyright © 2003 John Wiley & Sons, Ltd. 

Roessner U, Luedemann A, Brust D, *et al*. 2001. Metabolic profiling allows comprehensive phenotyping of genetically or environmentally modified plant systems. *Plant Cell* **13**: 11–29.

Schilling CH, Palsson BO. 2000. Assessment of the metabolic capabilities of *Haemophilus influenzae* Rd through a genome-scale pathway analysis. *J Theor Biol* **203**: 249–283.

Shaw AD, Kaderbhai N, Jones A, *et al*. 1999a. Non-invasive, on-line monitoring of the biotransformation by yeast of glucose to ethanol using dispersive Raman spectroscopy and chemometrics. *Appl Spectrosc* **53**: 1419–1428.

Shaw AD, Winson MK, Woodward AM, *et al*. 1999b. Rapid analysis of high-dimensional bioprocesses using multivariate spectroscopies and advanced chemometrics. *Adv Biochem Eng* **66**: 83–113.

Skolnick J, Fetrow JS, Kolinski A. 2000. Structural genomics and its importance for gene function analysis. *Nature Biotechnol* **18**: 283–287.

Smith TF. 1998. Functional genomics — bioinformatics is ready for the challenge. *Trends Genet* **14**: 291–293.

Tao H, Bausch C, Richmond C, Blattner FR, Conway T. 1999. Functional genomics: expression analysis of *Escherichia coli* growing on minimal and rich media. *J Bacteriol* **181**: 6425–6440.

Taylor J, Goodacre R, Wade WG, Rowland JJ, Kell DB. 1998. The deconvolution of pyrolysis mass spectra using genetic programming: application to the identification of some *Eubacterium* species. *FEMS Microbiol Lett* **160**: 237–246.

Taylor J, King RD, Altmann T, Fiehn O. 2002. Application of metabolomics to plant genotype discrimination using statistics and machine learning. *Bioinformatics* **18**(suppl 2): S241–248.

ter Kuile BH, Westerhoff HV. 2001. Transcriptome meets metabolome: hierarchical and metabolic regulation of the glycolytic pathway. *FEBS Lett* **500**: 169–171.

Thomas GH. 1999. Completing the *E. coli* proteome: a database of gene products characterized since the completion of the genome sequence. *Bioinformatics* **15**: 860–861.

Tiller PR, Land AP, Jardine I, *et al*. 2000. Characterization of metabolites by ion-trap LC–$MS^n$. *Am Biotechnol Lab* **18**: 58–79.

Timmins ÉM, Howell SA, Alsberg BK, Noble WC, Goodacre R. 1998. Rapid differentiation of closely related *Candida* species and strains by pyrolysis mass spectrometry and Fourier transform infrared spectroscopy. *J Clin Microbiol* **36**: 367–374.

Tjaden B, Saxena RM, Stolyar SI, *et al*. 2002. Transcriptome analysis of *Escherichia coli* using high-density oligonucleotide probe arrays. *Nucleic Acids Res* **30**: 3732–3738.

Tomita M, Hashimoto K, Takahashi K, *et al*. 1999. E-CELL: software environment for whole-cell simulation. *Bioinformatics* **15**: 72–84.

Tweeddale H, Notley-McRobb L, Ferenci T. 1998. Effect of slow growth on metabolism of *Escherichia coli*, as revealed by global metabolite pool ('metabolome') analysis. *J Bacteriol* **180**: 5109–5116.

Tweeddale H, Notley-McRobb L, Ferenci T. 1999. Assessing the effect of reactive oxygen species on *Escherichia coli* using a metabolome approach. *Redox Rep* **4**: 237–241.

Vaidyanathan S, Kell DB, Goodacre R. 2002. Rapid bacterial identification using flow-injection electrospray ionization mass spectrometry of whole cell extracts. *J Am Soc Mass Spectrom* **13**: 118–128.

Vaidyanathan S, Rowland JJ, Kell DB, Goodacre R. 2001. Rapid discrimination of aerobic endospore-forming bacteria via electrospray ionization mass spectrometry of whole cell suspensions. *Anal Chem* **73**: 4134–4144.

van Eijk HMH, Rooyakkers DR, Soeters PB, Deutz NEP. 1999. Determination of amino acid isotope enrichment using liquid chromatography–mass spectrometry. *Anal Biochem* **271**: 8–17.

Warne MA, Lenz EM, Osborn D, Weeks JM, Nicholson JK. 2000. An NMR-based metabonomic investigation of the toxic effects of 3-trifluoromethyl-aniline on the earthworm *Eisenia veneta*. *Biomarkers* **5**: 56–72.

Winson MK, Goodacre R, Timmins EM, *et al*. 1997. Diffuse reflectance absorbance spectroscopy taking in chemometrics (DRASTIC). A hyperspectral FT–IR-based approach to rapid screening for metabolite overproduction. *Anal Chim Acta* **348**: 273–282.

Winson MK, Todd M, Rudd BAM, *et al*. 1998. A DRASTIC (Diffuse Reflectance Absorbance Spectroscopy Taking in Chemometrics) approach for the rapid analysis of microbial fermentation products: quantification of Aristeromycin and Neplanocin A in *Streptomyces citricolor* broths. In *New Frontiers in Screening for Microbial Biocatalysts*, Kieslich K, van der Beek CP, de Bont JAM, van den Tweel WJJ (eds). Elsevier: Amsterdam; 185–191.

Wixon J, Kell DB. 2000. The *Kyoto Encyclopedia of Genes and Genomes* — KEGG: **http://www.genome.ad.jp/kegg**. *Yeast* **17**: 48–55.

Yanofsky C. 2000. Transcription attenuation: once viewed as a novel regulatory strategy. *J Bacteriol* **182**: 1–8.

Yanofsky C, Horn V. 1994. Role of regulatory features of the *trp* operon of *Escherichia coli* in mediating a response to a nutritional shift. *J Bacteriol* **176**: 6245–6254.

Yanofsky C, Konan KV, Sarsero JP. 1996. Some novel transcription attenuation mechanisms used by bacteria. *Posttranscriptional Control of Gene Expression: The Regulatory Role of RNA*. Elsevier: Hakone, Japan; 1017–1024.

Yanofsky C, Muh Ching Y, Horn V. 1993. Partial revertants of tryptophan synthetase $\alpha$-chain active site mutant Asp60–Asn. *J Biol Chem* **268**: 8213.

Copyright © 2003 John Wiley & Sons, Ltd. 
